# Identification and characterization of a novel fungal pyranose 2-oxidase from the *Punctularia strigosozonata* with potent cytotoxicity against pancreatic carcinoma cell lines

**DOI:** 10.3389/fmolb.2026.1914323

**Published:** 2026-10-28

**Authors:** Jiyang Guo, Junling Liu, Long Xu, Tingyi Yan, Zhendong Zheng

**Affiliations:** 1 Department of Oncology, General Hospital of Northern Theater Command, Shenyang, China; 2 School of Clinical Pharmacy, Shenyang Pharmaceutical University, Shenyang, China

**Keywords:** cytotoxicity, gene mining, heterologous expression, pancreatic cancer, pyranose oxidase

## Abstract

Pyranose oxidase (POx) is a FAD-dependent oxidoreductase that catalyzes the oxidation of D-glucose to 2-keto-D-glucose with concomitant production of hydrogen peroxide (H_2_O_2_). Although POx has been well characterized in several fungi and bacteria, its potential in cancer therapy remains largely unexplored. In this study, we identified a novel pyranose 2-oxidase, designated PsPOx, from the wood-decaying fungus *Punctularia strigosozonata* through gene mining. The gene was codon-optimized, synthesized, and heterologously expressed in *Escherichia coli*. The recombinant enzyme was purified and biochemically characterized. Sequence alignment and structural modeling confirmed that PsPOx possesses all conserved features of the GMC superfamily. Kinetic analysis showed that PsPOx exhibits the highest catalytic efficiency (*k*_cat_/*K*_m_ = 85.09 mM^-1^ s^-1^) towards D-glucose, with a *K*_m_ value of 0.8375 mM, indicating a strong substrate affinity. Using the CCK-8 assay, PsPOx demonstrated potent dose-dependent cytotoxicity against three human pancreatic carcinoma cell lines (PANC-1, AsPC-1, and BxPC-3), with IC50 values of 257.8, 195.2, and 176.2 ng mL^-1^, respectively. However, the IC_50_ value for the normal human pancreatic epithelial cell line hTERT-HPNE was 195.6 ng mL^-1^, which was comparable to those for AsPC-1 and BxPC-3, indicating that free PsPOx lacks selectivity between tumor and normal cells. Mechanistic studies revealed that PsPOx treatment increased intracellular and mitochondrial reactive oxygen species (ROS) levels, induced G0/G1 cell cycle arrest, downregulated Cyclin D1 and PCNA expression, and triggered apoptosis in BxPC-3 cells. These results preliminarily demonstrate that PsPOx possesses antitumor activity *in vitro* and may serve as a candidate for further development.

## 1 Introduction

Pyranose oxidase (POx; or named pyranose oxygen 2-oxidoreductase, EC 1.1.3.10) is a flavin adenine dinucleotide (FAD)-dependent oxidoreductase that belongs to the glucose-methanol-choline (GMC) superfamily (Wongnate and Chaiyen, 2013; Sützl et al., 2019; Abrera et al., 2020). Within the Carbohydrate-Active enZYme (CAZy) database (http://www.cazy.org/), POx is also classified under Auxiliary Activity (AA) family 3 (AA3) (Sützl et al., 2018), specifically in subfamily AA3_4 (other AA3 subfamilies include AA3_1 for cellobiose dehydrogenase, AA3_2 for aryl alcohol oxidase, glucose oxidase (GOx), and pyranose dehydrogenase, and AA3_3 for alcohol oxidases) (Sützl et al., 2018; Abrera et al., 2020). POx catalyzes the regioselective oxidation of aldopyranoses, preferentially D-glucose, at the C-2 position to yield 2-keto-D-glucose (Kujawa et al., 2006), while reducing molecular oxygen to hydrogen peroxide (H_2_O_2_) (Wongnate and Chaiyen, 2013; Abrera et al., 2020). The physiological role of POx is primarily associated with lignocellulose degradation in wood-degrading fungi, where it is secreted extracellularly and fuels peroxidases by generating H_2_O_2_ (Abrera et al., 2020).

Previously, POx was mainly characterized from fungal sources (Takakura and Kuwata, 2003; Bastian et al., 2005; Heckmann-Pohl et al., 2006; Dietrich and Crooks, 2009; Salaheddin et al., 2010; Pisanelli et al., 2012), such as TmPOx from Trametes ochracea (synonym *Trametes multicolor*) (Leitner et al., 2001; Martin Hallberg et al., 2004; Spadiut et al., 2008; 2009), PcPOx from *Phanerochaete chrysosporium* (Pisanelli et al., 2009; Hassan et al., 2013), LsPOx from *Lyophyllum shimeji* (Salaheddin et al., 2010) and AnPOx from *Aspergillus nidulans* (Pisanelli et al., 2012). Structurally, fungal POxs are homotetramers of approximately 270 kDa, with each monomer containing an FAD-binding domain featuring a Rossmann fold and a substrate-binding domain formed by a six-stranded central β-sheet and associated α-helices (Kostelac et al., 2022). The FAD cofactor is typically covalently linked to a histidine residue in each subunit (Kostelac et al., 2022). The catalytic mechanism follows a ping-pong bi-bi kinetics, comprising a reductive half-reaction, in which a hydride equivalent is transferred from the substrate to FAD, and an oxidative half-reaction, in which the reduced FADH_2_ is reoxidized by electron acceptors such as O_2_, quinones, or ferrocenium ions (Prongjit et al., 2009; Rungsrisuriyachai and Gadda, 2009). Recently, some bacterial POxs were mined and well characterized, revealing distinct properties compared to fungal POxs (Mendes et al., 2016; Zhang et al., 2018; Herzog et al., 2019; Kostelac et al., 2022; Virginia and Peterbauer, 2023; Santema et al., 2024). For instance, AsPOx from *Arthrobacter siccitolerans* (synonym *Pseudoarthrobacter siccitolerans*; PDB: 7QF8) (Mendes et al., 2016) is monomeric and contains non-covalently associated FAD, whereas KaPOx from *Kitasatospora aureofaciens* (synonym *Streptomyces aureofaciens*) (Virginia and Peterbauer, 2023) is dimeric, with FAD covalently linked to a histidine residue, similar to fungal POxs. Furthermore, some bacterial genes previously annotated as POx have been shown to encode C-glycoside 3-oxidase (CGOx) that preferentially oxidize glycosides rather than monosaccharides (Kostelac et al., 2022; 2024; 2025; Taborda et al., 2023; Santema et al., 2024). POx has been employed in both biosensor and biofuel cell applications (Abrera et al., 2020; Şahin et al., 2018; Kuenzel et al., 2020).

In recent years, the application of biocatalysis in cancer therapy has attracted widespread attention, with strategies including prodrug activation, reactive oxygen species (ROS) generation, depletion of essential nutrients in the tumor microenvironment, and drug release from antibody-drug conjugates (ADCs) (Liu et al., 2023). Among them, recombinant asparaginases (ASNases) from bacterial resources have been approved by the FDA as a first-line drug for the treatment of acute lymphoblastic leukemia (ALL) (Andjelkovic et al., 2025). Meanwhile, due to the metabolic reprogramming, tumor cells exhibit a preference for aerobic glycolysis (the Warburg effect), consuming approximately ten-fold more glucose than normal cells (Wang et al., 2026). Therefore, glucose oxidase (GOx), a member of the GMC family, has been widely formulated into nano-formulations capable of effectively inhibiting tumor growth by depleting glucose (Agrawal et al., 2024; Song et al., 2025; Wang et al., 2026). Similar to POx, GOx catalyzes the oxidation of D-glucose and also produces H_2_O_2_, which is subsequently converted to ROS (hydroxyl radicals), thereby inducing oxidative damage to tumor cells (Agrawal et al., 2024; Song et al., 2025; Wang et al., 2026).

However, the GOx is naturally derived from fungi and insects, with the majority being expressed by Penicillium and Aspergillus species (Bauer et al., 2022; Wang et al., 2022). Heterologous expression of GOx in *E. coli* (*Escherichia coli*) or other prokaryotic hosts has several limitations, including absent or negligible expression, low yields of soluble protein, and insufficient post-translational modification (Wang et al., 2022). With the same catalytic function, POx has been confirmed well expressed in *E. coli* in as mentioned previously, yet its anti-tumor potential remains largely unexplored (Ban et al., 2023). In addition, POx can accept both the α- and β-anomeric forms of D-glucose compared with only the β-anomer specific for GOx (Abrera et al., 2020). Moreover, POx overall exhibits a higher affinity for D-glucose, as its Michaelis constant (*K*_m_) is significantly lower than that of GOx (such as the *K*_m_ of TmPOx is 0.74 mM (Leitner et al., 2001), whereas that of AnGOx is 21.06 mM (Wang et al., 2022; Abrera et al., 2020). In summary, POx exhibits more advantages than GOx for the applications in biocatalysis and especially higher potential in tumor therapy or emerging live-bacteria therapy (Raman et al., 2023) based on synthetic biology.

In this study, we mined the genome of the fungus *Punctularia strigosozonata* and identified a novel pyranose oxidase, designated PsPOx. The gene was codon-optimized, heterologously expressed in *E. coli*, and the recombinant enzyme was purified and biochemically characterized. We further evaluated its *in vitro* cytotoxicity against three pancreatic cancer cell lines and a normal pancreatic epithelial cell line, and explored the underlying mechanisms by measuring intracellular and mitochondrial ROS levels, cell cycle distribution, apoptosis, and the expression of proliferation-related proteins.

## 2 Materials and methods

### 2.1 Chemicals and reagents

D-glucose, D-galactose, D-mannose, D-xylose, 2,2′-Azino-bis(3-ethylbenzthiazoline-6-sulfonic acid) (ABTS), horseradish peroxidase (HRP), isopropyl β-D-1-thiogalactopyranoside (IPTG), and reagents required for *E. coli* culture were purchased from Sangon Biotech (Shanghai, China). And the Cell Counting Kit-8 (CCK-8), BCA Protein Assay Kit and reagents required for cell culture were purchased from EallBio (Beijing, China). All other chemicals were of analytical grade.

### 2.2 Gene mining, sequence and structural analysis

The amino acid sequence of TmPOx (UniProt Q7ZA32) was used as a query to search the genome of *P. strigosozonata* using BLASTP of NCBI database (Sayers et al., 2021). Multiple sequence alignment was performed with Clustal Omega (Sievers et al., 2011). The tetrameric structure of PsPOx was predicted with AlphaFold3 (He et al., 2025), and modified by PyMOL (DeLano, 2002) to obtain Covalent His166-FAD in the active pocket of PsPOx based on the structure of TmPOx (PDB: 1TT0).

### 2.3 Cloning, expression and purification

Based on the amino acid sequence of PsPOx (NCBI accession number: XP_007389296.1), the PsPOx gene (Supplementary Table S1) was synthesized with codon-optimization for *E. coli* by Sangon Biotech (Shanghai, China). The synthesized gene was inserted into the *Nde*I/*Xho*I sites of pET-22b (+) with a C-terminal 6×His tag by Sangon Biotech. Then we transformed it into *E. coli* Rosetta (DE3) competent cells for protein expression. A positive transformant was inoculated into 10 mL of LB medium containing 50 μg mL^-1^ ampicillin and cultured at 37 °C overnight. The overnight culture was then diluted 1:100 into 500 mL of fresh LB medium containing 50 μg mL^-1^ ampicillin and grown at 37 °C until the OD_600_ reached 0.6–0.8. The culture was chilled on ice for 30 min and then induced with 0.1 mM IPTG at 20 °C overnight.

After induction, cells were harvested by centrifugation at 3000 rpm for 10 min, and the LB supernatant was discarded. The cell pellet was resuspended in Buffer A (100 mM NaPi, 500 mM NaCl, 20 mM imidazole, pH 7.0) at approximately 1 mL per 100 mg of wet cells. The cell suspension was kept on ice and disrupted by ultrasonication at 75 W with 5 s pulses and 5 s intervals for a total duration of 3 min. The lysate was then centrifuged at 4 °C, 12,000 rpm for 10 min, and the supernatant (crude enzyme extract) was collected for SDS-PAGE analysis and protein purification. The protein was purified by Ni-NTA column. In briefly, the supernatant was loaded onto a Ni-NTA column pre-equilibrated with Buffer A (100 mM NaPi, 500 mM NaCl, 20 mM imidazole, pH 7.0). After washing with Buffer A to remove non-specifically binding protein from the crude enzyme, the target protein was eluted with Buffer B (100 mM NaPi, 500 mM NaCl, 250 mM imidazole, pH 7.0). The eluted fractions were collected and desalted using a Millipore ultrafiltration tube (50 kDa). The protein was then resuspended and washed three times with 100 mM NaPi (pH 7.0, without imidazole) to ensure the removal of residual imidazole. Protein purity was assessed by SDS-PAGE, and concentration was determined by the BCA Protein Assay Kit (ZJ102, Epizyme, Shanghai, China).

### 2.4 Characterizations of PsPOx

#### 2.4.1 Enzyme activity assay

The PsPOx activity was measured spectrophotometrically using an HRP-ABTS coupled assay. The reaction mixture (200 µL) contained 100 mM NaPi (pH 8.0), 0.1 mM ABTS, 2 U mL^-1^ HRP, 5 mM monosaccharide, and appropriately diluted enzyme. All assays were performed at room temperature. The increase in absorbance at 405 nm (ε = 36.8 mM^−1^ cm^−1^) was monitored for 5 min. One unit (U) of activity was defined as the amount of enzyme producing 1 μmol H_2_O_2_ per minute. All assays were performed in triplicate.

#### 2.4.2 Optimum pH

The optimal pH of PsPOx was determined using the standard enzyme activity assay described above, except that different buffer systems were employed. The following buffers were used 100 mM of citrate buffer (pH 4.0 and 5.0), NaPi (pH 6.0, 7.0, and 8.0), and glycine–NaOH buffer (pH 9.0 and 10.0). All assays were performed in triplicate, and the activity was expressed as a percentage of the maximum activity observed.

#### 2.4.3 Optimum temperature

For determination of optimal Temperature, the glucose oxidation reaction was performed separately prior to the chromogenic detection in order to avoid the interference of elevated temperature on HRP in the coupled assay. A 200 µL reaction mixture containing 100 mM NaPi (pH 8.0), 5 mM D-glucose, and an appropriate amount of diluted enzyme was incubated at various temperatures (30 °C–70 °C, at 10 °C intervals) for 5 min. Then a 100 μL reaction mixture was added to 100 µL of chromogenic solution consisting of 2 mM ABTS and 1 U mL^-1^ HRP in 100 mM NaPi (pH 8.0). The increase in absorbance at 405 nm was recorded spectrophotometrically, reflecting the H_2_O_2_ produced during the initial glucose oxidation step. All assays were performed in triplicate, and the activity was expressed as a percentage of the maximum activity observed.

#### 2.4.4 Thermal stability

The thermal stability of PsPOx was evaluated by determining both the half-inactivation temperature and the half-life. For the half-inactivation temperature (T_50_^30^) determination, approximately 0.125 mg mL^-1^ purified enzyme in 100 mM NaPi (pH 8.0) were incubated in a PCR thermal cycler at a temperature gradient ranging from 30 °C to 70 °C for 30 min, immediately chilled on ice for 5 min, and the residual activity was measured under the standard assay conditions. The temperature at which 50% of the initial activity was lost was taken as the T_50_^30^. For the half-life determination, the enzyme solution (0.125 mg mL^-1^ in the same buffer) was incubated at 40, 45, and 50 °C. At predetermined time intervals, enzymes were cooled on ice for 5 min, and the residual activity was assayed as described above. The half-life (t_1/2_) at 50 °C was calculated from the activity decay curves. All assays were performed in triplicate, and the activity was expressed as a percentage of the maximum activity observed.

#### 2.4.5 Kinetic parameters

To determine kinetic parameters, initial velocities were measured at varying monosaccharide concentrations under the standard assay conditions and fitted to the Michaelis–Menten equation using nonlinear regression using GraphPad Prism software. All experiments were performed in triplicate.

### 2.5 Cell culture and cytotoxicity assay

The cytotoxic activity of PsPOx against three human pancreatic carcinoma cell lines (PANC-1, AsPC-1, and BxPC-3) and one normal human pancreatic epithelial cell line (hTERT-HPNE) was evaluated using the CCK-8 assay (03.S17002DB, EallBio, Beijing, China). Cells were cultured in appropriate medium supplemented with 10% fetal bovine serum (FBS) and 1% penicillin-streptomycin at 37 °C in a 5% CO_2_ humidified incubator. For the assay, cells in the logarithmic growth phase were seeded into 96-well plates at a density of 5 × 10^3^ cells per well (100 µL) and cultured for 24 h. Subsequently, the cells were treated for 24 h with PsPOx at concentrations ranging from 0 to 2000 ng mL^-1^ in triplicate, along with six control wells containing medium alone. After treatment, 10 µL of CCK-8 solution was added to each well, and incubated for 1 h at 37 °C. The absorbance was measured at 450 nm using a microplate reader. The half-maximal inhibitory concentration (IC_50_) was calculated by nonlinear regression analysis using GraphPad Prism software.

### 2.6 Measurement of intracellular and mitochondrial ROS

Intracellular ROS and mitochondrial superoxide levels were measured using the Reactive Oxygen Species Assay Kit (KGA7308, KeyGEN, Nanjing, China) and MitoSOX Red (V36519, Yuanye, Shanghai, China), respectively. BxPC-3 cells were seeded (10^6^) in 60 mm Petri plates and cultured 24 h. The cells were then treated with PsPOx at 0, 176.2, and 352.4 ng mL^-1^ for 24 h. After treatment, cells were collected by trypsinization, washed twice with PBS, and resuspended in PBS at a density of approximately 1 × 10^6^ cells mL^-1^. For intracellular ROS detection, cells were incubated with 4 μM DCFH-DA at 37 °C for 20 min in the dark, washed, and resuspended in PBS. For mitochondrial superoxide detection, cells were incubated with 1 μM MitoSOX Red at 37 °C for 20 min in the dark, washed, and resuspended in PBS. Fluorescence was analyzed by flow cytometry using FITC (DCFH-DA) and PE (MitoSOX Red) channels, with at least 10,000 events acquired per sample.

### 2.7 Cell cycle analysis

BxPC-3 cells (4 × 10^5^) were seeded in 6-well plates and incubated at 37 °C for 24 h. After the treatment with PsPOx at 0, 176.2, and 352.4 ng mL^-1^ for 24 h, Cells were collected, washed with PBS, and fixed in 70% cold ethanol at −20 °C overnight. After centrifugation, cells were washed with PBS and stained with PI/RNase A working solution (KGA9101, KeyGEN) at room temperature for 30 min in the dark. Cell cycle distribution was analyzed by flow cytometry with at least 10,000 events acquired per sample.

### 2.8 Determination of proliferation-related protein expression after PsPOx treatment

Expression of proliferation-related proteins, including Cyclin D1 and PCNA, was determined in control and PsPOx-treated BxPC-3 cells by immunoblot assay. A protease inhibitor cocktail (02.11021, EallBio, Beijing, China) and ice-cold RIPA lysis solution (02.12264–20, EallBio) were used to lyse the treated cells. The protein content in the lysates was measured using the BCA Protein Assay Kit (ZJ102, Epizyme, Shanghai, China). The protein (20 μg) was electroblotted onto the PVDF membrane after being resolved on 10% SDS-PAGE. After blotting, 5% skimmed milk was used to incubate the membrane for 1 h. This was followed by primary antibody incubation; anti-Cyclin D1 (Abcam, AB134175, 1:1000), anti-PCNA (Abcam, AB92552, 1:1000), and anti-α-tubulin (Abcam, AB52866, 1:5000) at 4 °C overnight. After cleaning, the membrane was incubated for 1 h with the appropriate horseradish peroxidase-conjugated secondary antibody (Abcam, 1:5000). After washing, bands of specific proteins were detected with an ECL chemiluminescence detection reagent, visualized using an ECL imager, quantified using ImageJ software, and statistical analysis was performed.

### 2.9 Evaluation of induction of apoptosis/necrosis after PsPOx treatment

Apoptosis in BxPC-3 cells by PsPOx treatment was evaluated by a double dye method using Annexin V-FITC and propidium iodide. BxPC-3 cells (4 × 10^5^) were seeded in 6-well plates and incubated at 37 °C for 24 h. After the treatment with PsPOx at 0, 176.2, and 352.4 ng mL^-1^ for 24 h, cells were collected by digestion with EDTA-free trypsin and washed twice with PBS. Apoptosis was evaluated using an Annexin V-FITC/PI Apoptosis Detection Kit (KGA1102, KeyGEN) according to the manufacturer’s instructions. The samples were subjected to flow cytometry analysis. Data were analyzed using FlowJo software. Cells negative for both Annexin V-FITC and PI were considered viable, Annexin V^+^/PI^−^ cells were considered early apoptotic, and Annexin V^+^/PI^+^ were considered late apoptotic. The total apoptotic rate was calculated as the sum of early and late apoptotic cells. All experiments were performed in triplicate.

## 3 Result

### 3.1 Gene mining and heterologous expression

To discover a novel pyranose oxidase, we mined the genome of the wood-decaying fungus *P. strigosozonata* using the amino acid sequence of TmPOx as a query for BLASTP by using the NCBI database. A full-length open reading frame encoding a putative POx (named PsPOx, XP_007389296.1) of 606 amino acids was identified, showing 94% query coverage with TmPOx. After synthesizing the PsPOx gene and constructing the recombinant *E. coli* strain, the heterologous expression of PsPOx was evaluated by SDS-PAGE. Although the recombinant protein was predominantly present in the insoluble fraction as inclusion bodies, a prominent soluble band at the expected monomeric molecular weight of 66.9 kDa (theoretical) was also observed (Supplementary Figure S1) and subsequently purified. The soluble fraction was purified by Ni-NTA affinity chromatography. The purification process yielded a soluble expression level of 5.38 mg L^-1^ of culture, with a purification yield of 33.1% and a purification fold of 7.67 based on purity. The specific activity of the purified PsPOx was determined to be 0.9705 U mg^-1^ (Table 1). These results indicate that although a considerable portion of the recombinant protein was insoluble, the soluble fraction could be efficiently purified to obtain active PsPOx suitable for subsequent biochemical characterization and functional assays.

**TABLE 1:** Specific activity and kinetic parameters of PsPOx.

Monosaccharides	Specific activity (U mg^-1^)	*K*_m_ (mM)	*k*_cat_ (min^-1^)	*k*_cat_/*K*_m_ (mM^-1^ min^-1^)
D-glucose	0.9705	0.8375 ± 0.044	71.26 ± 1.37	85.0866
D-galactose	0.0234	5.201 ± 0.305	3.335 ± 0.056	0.6412
D-xylose	0.1031	26.58 ± 1.09	42.3 ± 0.78	1.5914
D-Mannose	0.0283	125.6 ± 5.0	61.26 ± 1.0	0.4877
1,5-Anhydroglucitol	0.1660	18.78 ± 2.02	43.45 ± 1.68	2.3136

### 3.2 Sequence and structural analysis

Following successful expression, PsPOx was characterized by multiple sequence alignment with previously reported fungal POxs. PsPOx shares 45.61%–54.96% sequence identity with four reported fungal POxs (Supplementary Table S2). As shown in Figure 1, PsPOx exhibits all the hallmark features of the GMC (glucose–methanol–choline) oxidoreductase family, particularly those characteristics of fungal pyranose 2-oxidases. The primary structure of PsPOx contains the conserved ADP-binding βαβ-motif (Rossmann fold) typical of FAD-dependent oxidoreductases. Notably, the FAD-attachment motif, identified as ^165^STHW^168^ in TmPOx and responsible for covalent 8α-(N3)-histidyl flavinylation via His167, is conserved in PsPOx (^164^TTHW^167^, His166 in Figure 2b) (Martin Hallberg et al., 2004). The essential catalytic His–Asn pair (H548 and N593 in TmPOx), located at the re-face of the isoalloxazine ring, is fully conserved in PsPOx (H539 and N582, Figure 2b), indicating a shared mechanism for substrate oxidation. Furthermore, sequence alignment reveals that PsPOx contains the putative oligomerization loop and arm regions (residues 102–158 in PsPOx; 108–158 in TmPOx), which mediate homotetramer formation and stabilize the internal cavity (Figure 2a). The active-site loop (residues 452–461 in TmPOx), which in its closed conformation blocks substrate access and must undergo a conformational change for catalysis, is also present in PsPOx (residues 444–455), albeit with modest sequence variation. Moreover, the residues T168, Q440, D444, Y448, and A537 in active pocket are responsible for substrate recognition based on the PcPOx (Hassan et al., 2013). Additionally, the head domain (residues 368–430 in TmPOx), a structurally unique αββα motif protruding from the central body, appears to be conserved in PsPOx (residues 382–422), suggesting a potential role in oligomerization or interaction with the fungal cell wall. Consequently, these sequence features confirm that PsPOx is a novel fungal pyranose 2-oxidase that retains the tetrameric organization, covalently bound FAD, and the essential catalytic machinery required for H_2_O_2_ generation during lignocellulose degradation.

**FIGURE 1:**
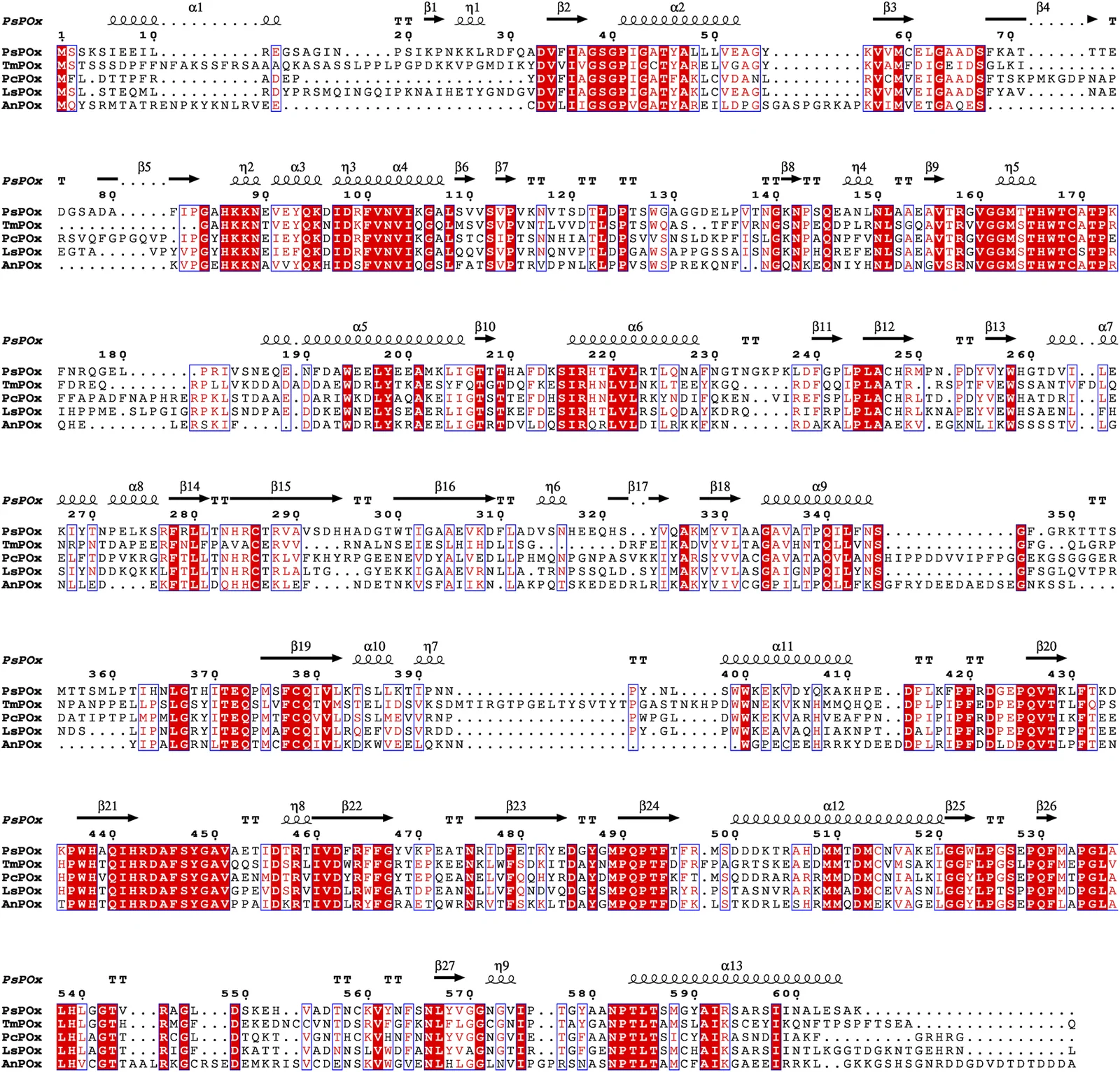
Multiple sequence alignment of PsPOx with fungal pyranose oxidases, including TmP2Ox (PDB: 1TT0), PcPOx (PDB: 4MIF), LsPOx (Uniprot Q75ZP8) and AnPOx (Uniprot Q5B2E9). The visualization was generated using the ESPript 3.2 web server (Robert et al., 2025) with the secondary structure annotation based on the AlphaFold 3-predicted model of PsPOx.

**FIGURE 2:**
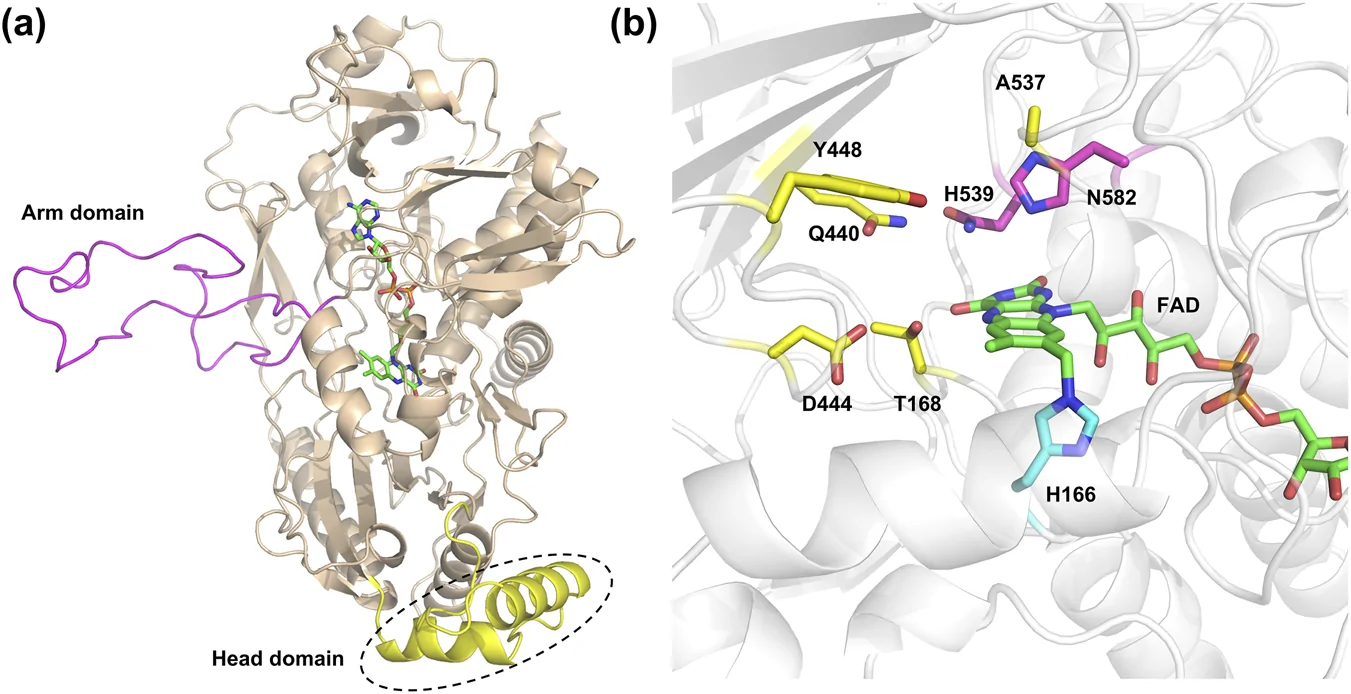
**(a)** Monomeric structure of PsPOx predicted by AlphaFold 3 model of PsPOx. **(b)** Amino acid distribution analysis of active pocket of PsPOx. The visualization was generated using the PyMOL.

### 3.3 Biochemical characterization

The biochemical characteristics of the pyranose oxidase PsPOx were systematically investigated. As shown in Figure 3a, the enzyme displayed a clear pH-dependent activity profile, with the maximum activity observed at pH 8.0. The relative activity decreased sharply under acidic conditions (below pH 5.0) and also dropped notably at pH 9.0 and 10.0, indicating that PsPOx functions optimally in a slightly alkaline environment. The optimum temperature was determined by measuring the activity at various temperatures ranging from 30 °C to 70 °C (Figure 3b). PsPOx exhibited the highest activity at 35 °C, and activity dropped rapidly at temperatures beyond 45 °C, suggesting that the enzyme is moderately thermophilic. To further evaluate thermal stability, the enzyme was incubated at different temperatures (30 °C–70 °C) for 30 min, and the residual activity was measured (Figure 3c). The temperature at which 50% of the initial activity was lost (T_50_^30^) was calculated by curve fitting to be 62.1 °C, reflecting the enzyme’s resistance to heat inactivation. Additionally, the thermal inactivation kinetics were examined at three temperatures (40, 45, and 50 °C) over time (Figure 3d). The residual activity decreased gradually with longer incubation times. PsPOx remains 78.2% and 62.6% activity at 40 °C and 45 °C, respectively. As for at 50 °C, PsPOx lost about 82.1% of its activity within 10 h, and the half-life (t_1/2_) was determined to be 4.68 h, indicating that the enzyme retains considerable activity under moderately high temperature conditions for several hours.

**FIGURE 3:**
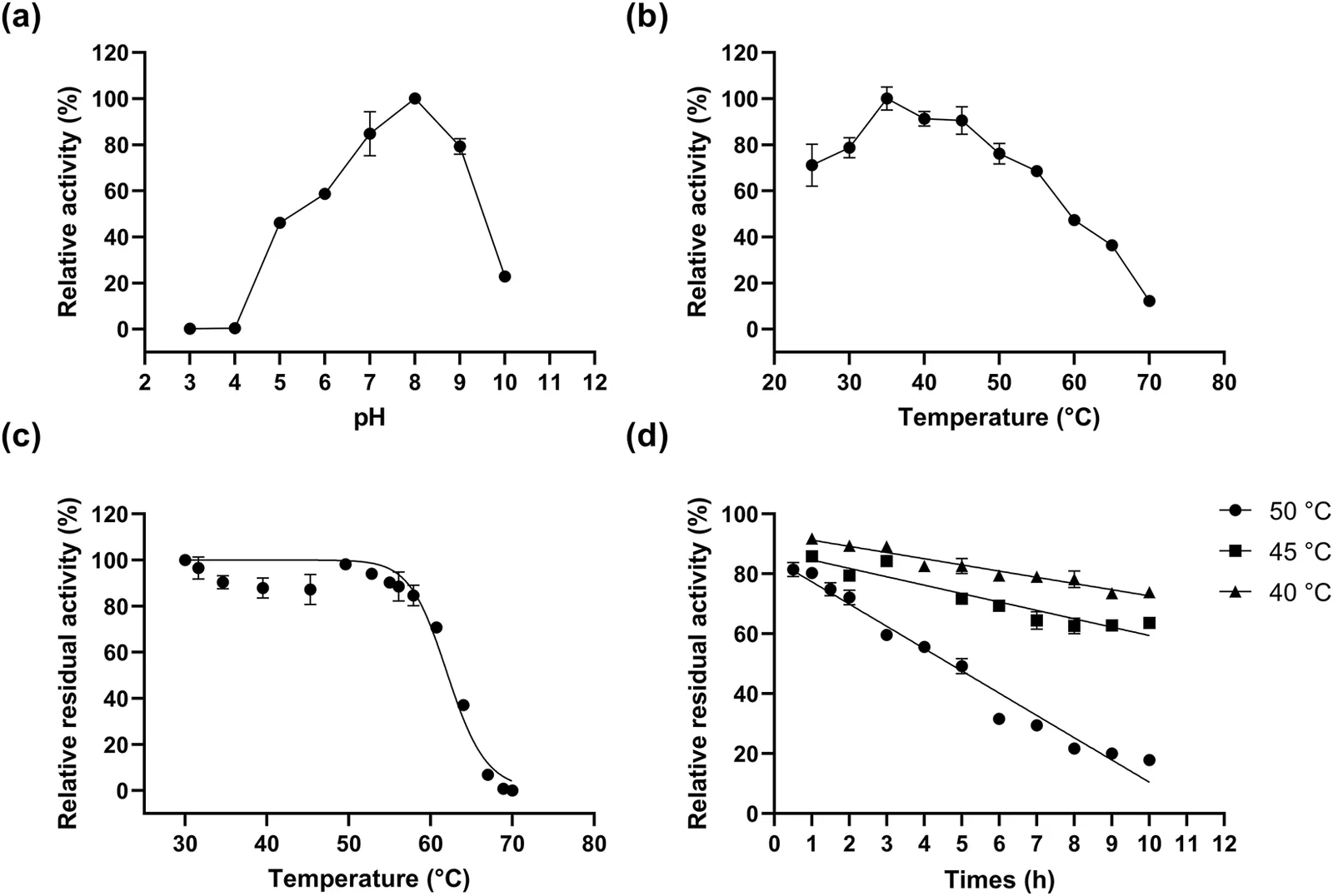
Biochemical characterization of PsPOx. **(a)** Effect of pH on enzyme activity. The activity measured at pH 8.0 was defined as 100% relative activity. **(b)** Effect of temperature on enzyme activity. The activity measured at 35 °C was defined as 100% relative activity. **(c)** Determination of the half-inactivation temperature (T_50_^30^). **(d)** Determination of the half-lives at 40, 45, and 50 °C. For both **(c,d)**, the residual activity was measured relative to that of the unincubated enzyme (set as 100%).

### 3.4 Substrate specificity and kinetic parameters

The specific activity and kinetic parameters of PsPOx towards four common monosaccharides were determined and are summarized in Table 1 and Supplementary Figure S2. Among the tested substrates, PsPOx exhibited the highest specific activity (0.9705 U mg^-1^) and catalytic efficiency (*k*_cat_/*K*_m_ = 85.09 mM^-1^ min^-1^) towards D-glucose, with a *K*_m_ value of 0.8375 mM, indicating a high affinity for this nature substrate. In contrast, the specific activities for D-galactose, D-xylose, and D-mannose were substantially lower (0.0234, 0.1031, and 0.0283 U mg^-1^, respectively). Notably, although the turnover number (*k*_cat_) for D-mannose (61.26 min^-1^) was close to that for D-glucose (71.26 min^-1^), the extremely high *K*_m_ of 125.6 mM resulted in a catalytic efficiency of only 0.4877 mM^-1^ min^-1^, approximately 175-fold lower than that for glucose. D-Xylose displayed a moderate *k*_cat_ (42.3 min^-1^) but a relatively high *K*_m_ (26.58 mM), yielding a low overall efficiency. 1,5-Anhydroglucitol (1,5-AG) is a naturally occurring monosaccharide as a valuable non-traditional biomarker for diabetes monitoring (Xu et al., 2024a; 2024b). Its detection can be performed by mass spectrometry or enzymatic assays by pyranose oxidase. PsPOx displayed a specific activity of 0.1660 U mg^-1^, a *k*_cat_/*K*_m_ of 18.78 mM, and a catalytic efficiency of 2.31 mM^-1^ min^-1^, ranking it as the second most efficient substrate after D glucose among those tested. The affinity of PsPOx for 1,5-AG (*K*_m_ = 18.78 mM) was similar to that of PcPOx (*K*_m_ = 18.9 mM (Fujii et al., 2020)) and higher than that of CvPOx (*K*_m_ = 35.3 mM (Masuda-Nishimura et al., 1999)).

### 3.5 Cytotoxicity against pancreatic cancer cell lines and normal pancreatic epithelial cells

Given the high specificity and catalytic efficiency of PsPOx toward D-glucose, this enzyme may exert antitumor effects by efficiently depleting glucose, a critical nutrient for rapid proliferation of tumor cells. Such glucose-consuming activity provides a mechanistic basis for evaluating the potential anticancer properties of PsPOx *in vitro*. Therefore, to assess the antitumor potential of PsPOx, the cytotoxic activity of PsPOx was evaluated against three human pancreatic carcinoma cell lines (PANC-1, AsPC-1, and BxPC-3) and one normal human pancreatic epithelial cell line (hTERT-HPNE) using the CCK-8 assay. As shown in Figure 4; Supplementary Table S3, PsPOx exhibited significant cytotoxicity towards all three pancreatic cancer cell lines in a dose-dependent manner. Among them, BxPC-3 cells were the most sensitive to PsPOx treatment, with an IC_50_ value of 176.2 ng mL^-1^. AsPC-1 cells showed moderate sensitivity with an IC_50_ value of 195.2 ng mL^-1^, while PANC-1 cells were the least sensitive, with an IC_50_ value of 257.8 ng mL^-1^. Notably, the normal pancreatic epithelial cell line hTERT-HPNE also exhibited considerable sensitivity to PsPOx, with an IC_50_ value of 195.6 ng mL^-1^, which was comparable to that of AsPC-1 and BxPC-3 cells. These results demonstrate that PsPOx not only possesses potent cytotoxic activity against pancreatic carcinoma cells but also affects normal pancreatic epithelial cells under the present *in vitro* conditions, indicating that its tumor selectivity remains limited.

**FIGURE 4:**
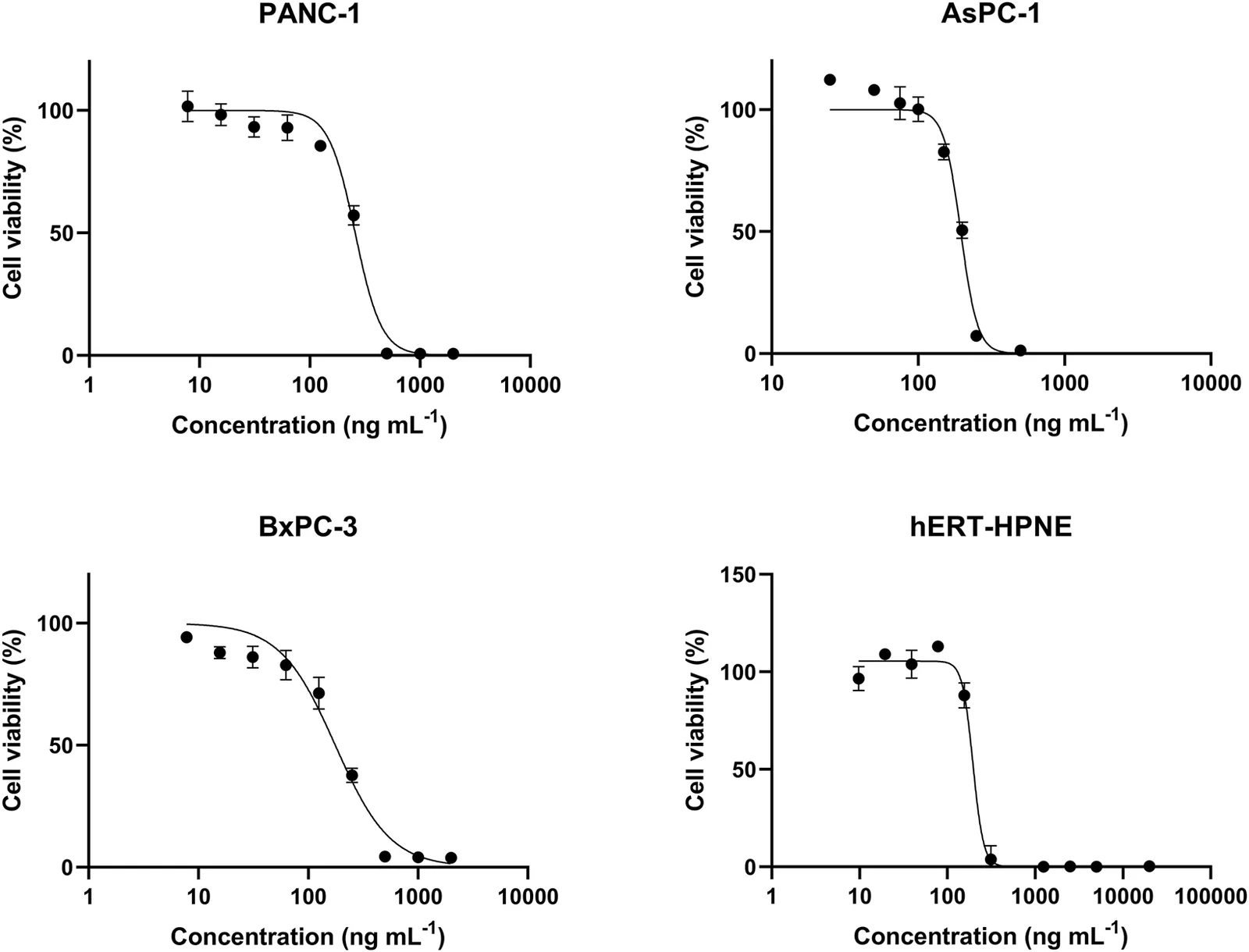
Dose-dependent inhibition of pancreatic cancer cells and normal pancreatic epithelial cells viability by pyranose oxidase.

### 3.6 Estimation of reactive oxygen species (ROS) production after PsPOx treatment

To investigate whether the cytotoxicity of PsPOx was associated with oxidative stress, intracellular ROS and mitochondrial superoxide levels were measured in BxPC-3 cells after treatment with PsPOx at 0, 176.2, and 352.4 ng mL^-1^. As shown in Figure 5a, flow cytometric analysis using DCFH-DA revealed a dose-dependent increase in intracellular ROS levels. The percentage of DCF-positive cells increased from 12.4% in the control group to 22.5% and 27.3% after treatment with 176.2 and 352.4 ng mL^-1^ PsPOx, respectively (Figure 5a). In addition, MitoSOX Red staining showed a more pronounced increase in mitochondrial superoxide generation (Figure 5b). The percentage of MitoSOX-positive cells increased from 18.7% in the control group to 52.8% and 65.7% at 176.2 and 352.4 ng mL^-1^, respectively. These results indicate that PsPOx treatment induces both intracellular ROS accumulation and mitochondrial superoxide production in pancreatic cancer cells. Since PsPOx catalyzes the oxidation of D-glucose to 2-keto-D-glucose with concomitant generation of H_2_O_2_, part of the intracellular ROS increase may come from extracellular H_2_O_2_ that diffuses into the cells. Collectively, these data support the hypothesis that PsPOx exerts its cytotoxic effect against pancreatic cancer cells through glucose depletion and H_2_O_2_/ROS-mediated oxidative stress.

**FIGURE 5:**
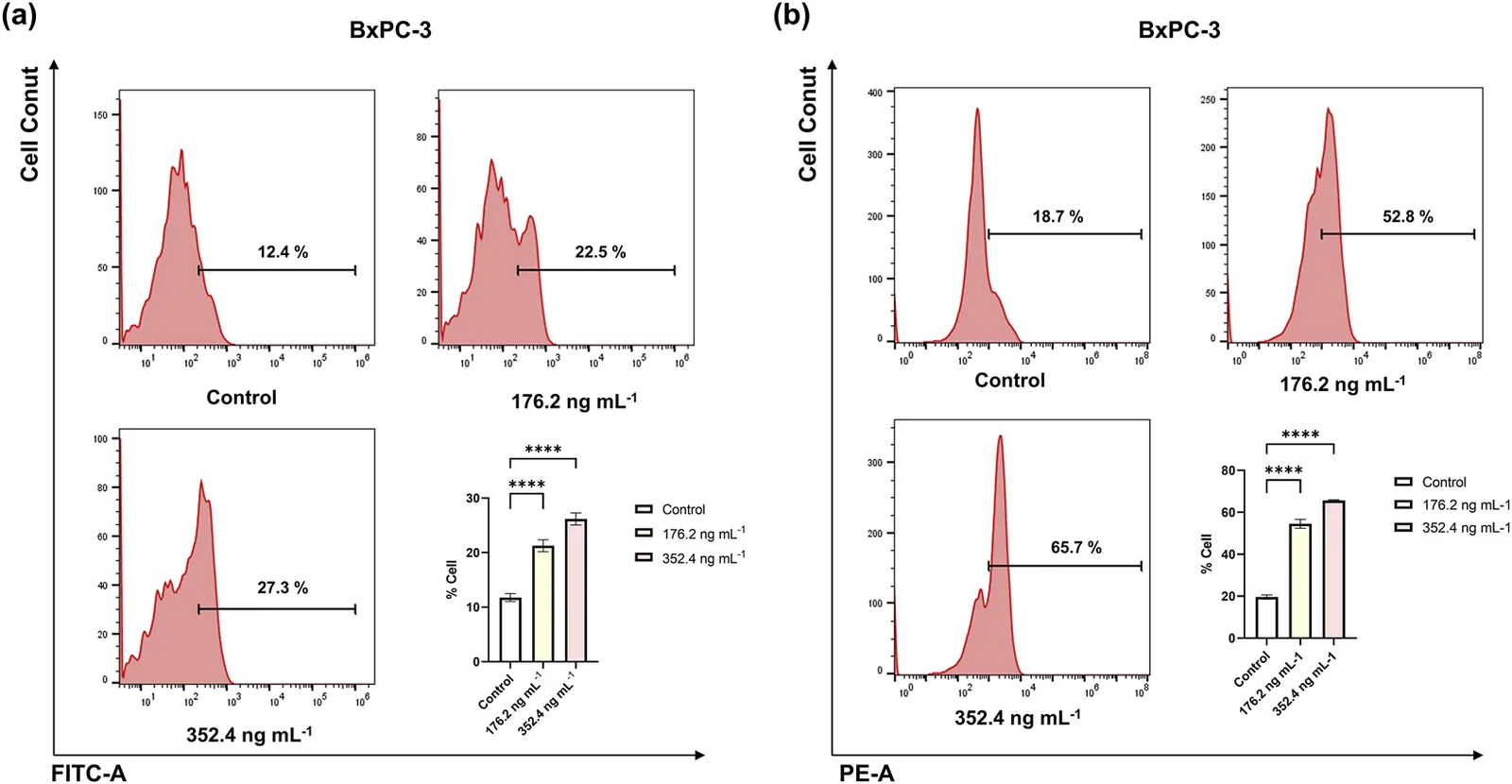
PsPOx induces **(a)** intracellular ROS and **(b)** mitochondrial ROS in BxPC-3 cells.

### 3.7 Cell cycle analysis after PsPOx treatment

To further investigate the anti-proliferative mechanism of PsPOx, cell cycle distribution was analyzed. As shown in Figure 6a, PsPOx induced a dose-dependent increase in the proportion of cells in the G0/G1 phase, accompanied by a decrease in the S phase. In the control group, the percentages of cells in G0/G1, S, and G2/M phases were 38.3%, 34.7%, and 25.9%, respectively. After treatment with 176.2 ng mL-1 PsPOx, the G0/G1 fraction increased to 72.4%, while the S and G2/M fractions decreased to 18.5% and 7.22%, respectively. At 352.4 ng mL^-1^, the G0/G1 fraction further increased to 76.2%, with S and G2 fractions at 11.6% and 11.1%, respectively. These results indicate that PsPOx induces G0/G1 cell cycle arrest in pancreatic cancer cells, thereby inhibiting cell proliferation.

**FIGURE 6:**
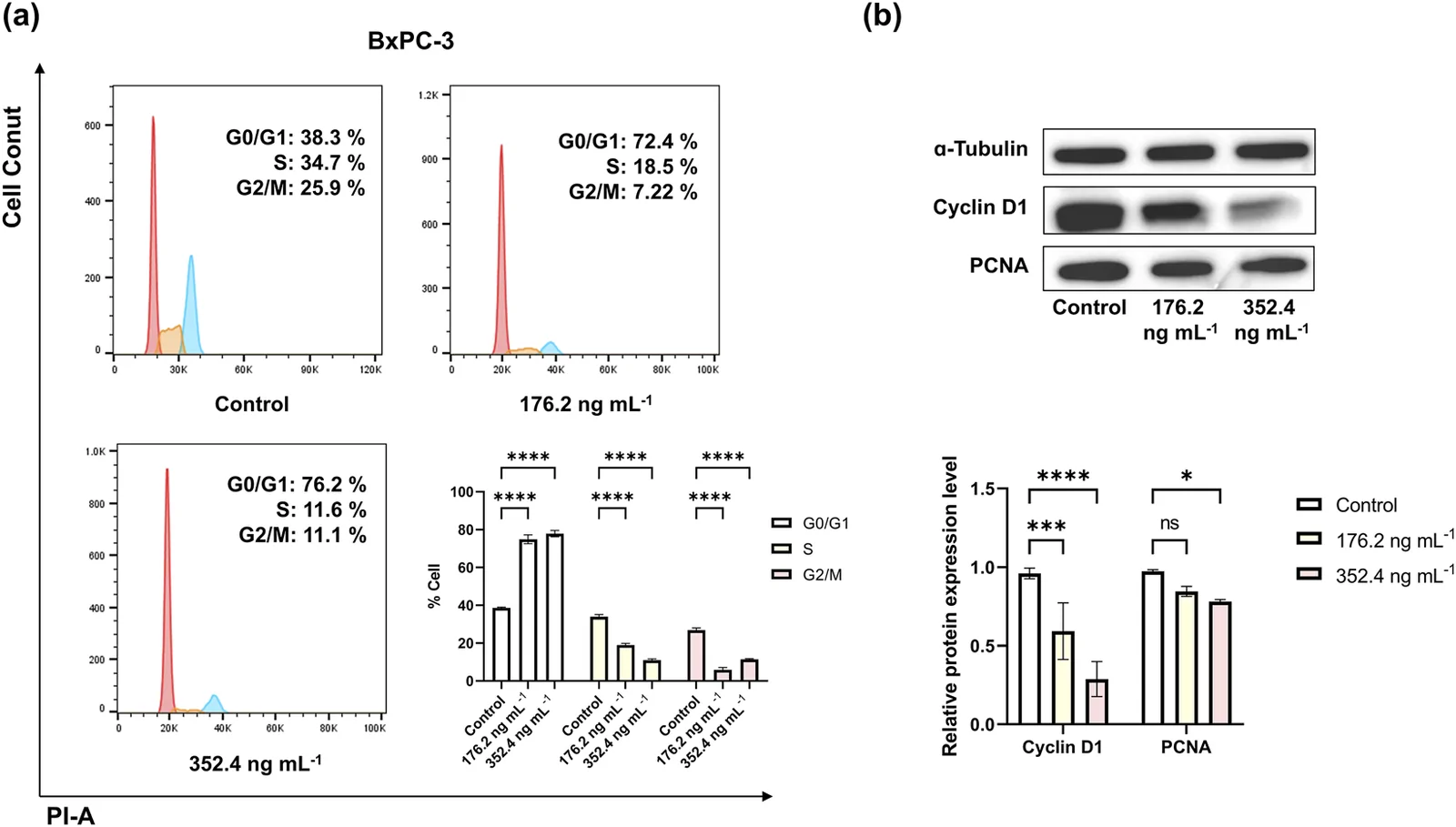
**(a)** Effect of PsPOx on different phases of cell cycle. **(b)** Western blot analysis revealed downregulated expression of Cyclin D1 and PCNA in BxPC-3 cells.

### 3.8 Western blot analysis of cyclin D1 and PCNA expression

To further confirm the anti-proliferative effect of PsPOx and its association with G0/G1 cell cycle arrest, the protein expression levels of Cyclin D1 and PCNA were examined by Western blotting. As shown in Figure 6b, PsPOx treatment resulted in a dose-dependent decrease in both Cyclin D1 and PCNA protein levels compared with the control group. In contrast, the expression of α-tubulin remained stable across all groups, confirming equal protein loading. Densitometric analysis further confirmed that the relative expression levels of Cyclin D1 and PCNA were significantly reduced in PsPOx-treated cells compared with the control group (P < 0.05). These results are consistent with the cell cycle analysis, in which PsPOx induced G0/G1 phase arrest and reduced the S phase population. Collectively, these data suggest that PsPOx inhibits pancreatic cancer cell proliferation by downregulating key proliferation-related proteins, including Cyclin D1 and PCNA.

### 3.9 Evaluation of induction of apoptosis/necrosis after treatment with PsPOx

To confirm that the cytotoxic effect of PsPOx was mediated by apoptosis, Annexin V-FITC/PI double staining was performed. As shown in Figure 7, PsPOx induced a dose-dependent increase in the apoptotic cell population. In the control group, the percentages of early apoptotic (Annexin V-FITC^+^/PI^−^) and late apoptotic (Annexin V-FITC^+^/PI^+^) cells were 3.16% and 4.88%, respectively, with a total apoptotic rate of approximately 8.0%. After treatment with 176.2 ng mL^-1^, PsPOx, the early and late apoptotic populations increased to 3.68% and 19.1%, respectively, resulting in a total apoptotic rate of approximately 22.8%. At 352.4 ng mL^-1^, the late apoptotic population markedly increased to 76.2%, with an early apoptotic population of 9.19%, yielding a total apoptotic rate of approximately 85.4% (Figure 7). In contrast, the percentage of necrotic cells (Annexin V-FITC^-^/PI^+^) remained low in all groups (0.11%, 0.19%, and 0.36% for control, 176.2, and 352.4 ng mL^-1^, respectively). These results indicate that PsPOx predominantly induces apoptosis, rather than necrosis, in pancreatic cancer cells, further supporting its potential as an anticancer agent.

**FIGURE 7:**
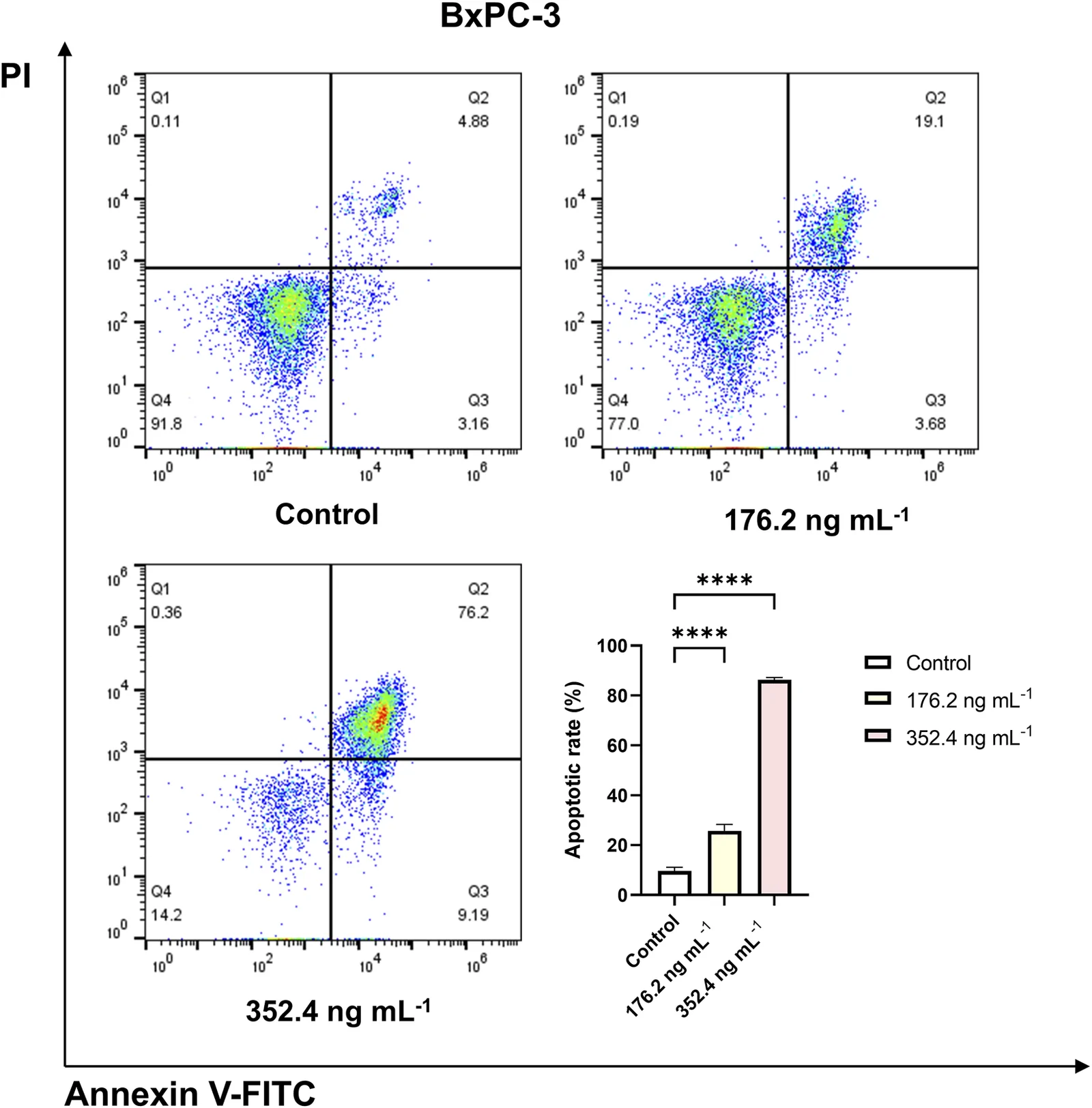
Flow cytometric analysis of BxPC-3 cell line after treatment with PsPOx and staining with Annexin-V/PI.

## 4 Discussion

In this study, we successfully identified, heterologously expressed, and characterized a novel pyranose 2-oxidase, PsPOx, from the fungus *P. strigosozonata*. Sequence and structural analyses confirmed that PsPOx belongs to the GMC oxidoreductase superfamily and retains all conserved features of fungal POxs, including the FAD-binding Rossmann fold, the covalent flavinylation motif, the catalytic His-Asn pair (H539 and N582), and the putative oligomerization loop and arm regions. These features strongly support a tetrameric organization and a catalytic mechanism analogous to that of well-studied fungal POxs such as TmPOx (Martin Hallberg et al., 2004) and PcPOx (Hassan et al., 2013).

Biochemical characterization revealed that PsPOx functions optimally at pH 8.0 °C and 35 °C, with a half-inactivation temperature (T_50_^30^) of 62.1 °C and a half-life of 4.68 h at 50 °C. In an earlier work by Dietmar group, they reported that the half-lives at 50 °C of three fungal POx LsPOx, TmPOx and PcPOx were 10.5 min, 70.3 min and 96 h respectively (Salaheddin et al., 2010). Compared to fungal-derived POx, bacterial-derived POx exhibits poorer thermal stability, which may be related to the non-covalent binding of FAD (Mendes et al., 2016; Zhang et al., 2018; Kostelac et al., 2022). For example, ScPOx exhibited a T_50_^30^ of 38 °C and a half-life of 2 min at 50 °C (Kostelac et al., 2022); PaPOx has a T_50_^30^ of about 50 °C (Zhang et al., 2018); and AsPOx has a half-life of only 25 min at 40 °C (Mendes et al., 2016). These properties indicate that PsPOx is moderately thermostable and operates efficiently under mildly alkaline conditions. Kinetic analysis demonstrated that PsPOx has a strong preference for D-glucose, with a *K*_m_ of 0.8375 mM, which is substantially lower than that of glucose oxidase (GOx, e.g., 21.06 mM for AnGOx (Wang et al., 2022)).

The cytotoxic activity of PsPOx was evaluated against three human pancreatic carcinoma cell lines and one normal human pancreatic epithelial cell line. PsPOx reduced cell viability in a dose-dependent manner towards all the cell lines. However, the IC_50_ for hTERT-HPNE cells was comparable to those for AsPC-1 and BxPC-3. This result indicates that free PsPOx does not exhibit selectivity between pancreatic cancer cells and normal pancreatic epithelial cells under the present *in vitro* conditions. Fortunately, glucose oxidase, which shares the same antitumor mechanism as pyranose oxidase and similarly suffers from poor targeting, has been extensively formulated into nanomedicines for tumor-targeted therapy, and various delivery strategies have been developed to improve its stability, tumor accumulation, and selectivity(Fu et al., 2018; 2019; 2020; 2023; Song et al., 2025; Wang et al., 2026). Given the similar catalytic mechanism of PsPOx and GOx, encapsulating PsPOx into rationally designed nanocarriers may help overcome its lack of targeting and improve its therapeutic index.

Mechanistic studies showed that PsPOx treatment increased intracellular ROS and mitochondrial superoxide levels, induced G0/G1 cell cycle arrest, downregulated Cyclin D1 and PCNA expression, and triggered apoptosis in BxPC-3 cells. These results are consistent with the generation of H_2_O_2_ during glucose oxidation and suggest that PsPOx can induce oxidative stress and inhibit proliferation in pancreatic cancer cells. However, these experiments were performed only *in vitro*, and the observed effects cannot be directly extrapolated to *in vivo* tumors. The tumor microenvironment is complex and heterogeneous, with varying glucose levels, oxygen tension, pH, and antioxidant capacity. Moreover, the pharmacokinetics, biodistribution, and systemic toxicity of PsPOx were not evaluated in this study.

## 5 Conclusion

In conclusion, we successfully identified a novel, biochemically robust fungal pyranose 2-oxidase, PsPOx from the fungus *P. strigosozonata*. The enzyme exhibits all conserved structural and catalytic features of the GMC superfamily. PsPOx shows high substrate specificity and catalytic efficiency toward D-glucose, with a low *K*_m_ value, making it an efficient glucose-depleting enzyme. Furthermore, *in vitro* cytotoxicity assays showed that PsPOx reduced the viability of pancreatic cancer cell lines in a dose-dependent manner. However, the enzyme also affected normal pancreatic epithelial cells to a similar extent, indicating a lack of tumor selectivity in its free form. Therefore, this work preliminarily demonstrates that PsPOx possesses antitumor activity *in vitro* and holds promise as a therapeutic agent. Further studies, particularly *in vivo* evaluations and targeted delivery strategies, are warranted to validate its therapeutic potential.

## Data Availability

The original contributions presented in the study are included in the article/Supplementary Material, further inquiries can be directed to the corresponding author.
