# Supplementary material for: Identification and characterization of a novel fungal pyranose 2-oxidase from the Punctularia strigosozonata with potent cytotoxicity against pancreatic carcinoma cell lines
Source: Front Mol Biosci. 2026 Oct 28;13:1914323. doi: 10.3389/fmolb.2026.1914323 (PMC13752094; doi:10.3389/fmolb.2026.1914323)
Supplement: Supplementary file 1 [file DataSheet1.docx]

*Supporting information*

**Identification and Characterization of a Novel Fungal Pyranose 2-Oxidase from the** ***Punctularia Strigosozonata* with Potent Cytotoxicity Against Pancreatic Carcinoma Cell Lines**

Jiyang Guo, ^1,^^‡^ Junling Liu, ^1,‡^ Long Xu, ^1^ Tingyi Yan,^1,2^ and Zhendong Zheng^*,1^

^1^Department of Oncology, General Hospital of Northern Theater Command, Shenyang 110016, China.

^2^ School of Clinical Pharmacy, Shenyang Pharmaceutical University, 103 Wenhua Road, Shenhe, Shenyang 110016, China.

‡ J.L. and J.G. contributed equally.

*Corresponding author: gcp_zzd@sina.com.

Table 1. The gene sequence of PsPOx.

ATGTCTTCAAAAAGTATTGAAGAGATACTAAGAGAAGGTAGCGCGGGTATCAACCCGTCGATCAAACCGAATAAGAAGCTGCGCGACTTCCAGGCGGATGTTTTTATCGCGGGCAGCGGTCCGATTGGTGCGACCTACGCGTTGCTGCTGGTCGAGGCTGGTTACAAAGTGGTAATGTGCGAATTGGGCGCTGCGGACAGCTTCAAAGCGACTACCACGGAGGACGGTTCCGCGGACGCGTTCATCCCAGGTGCGCATAAAAAAAACGAGGTCGAATACCAGAAGGACATTGACCGTTTCGTGAATGTGATTAAAGGTGCTCTCTCCGTGGTTAGCGTGCCGGTTAAGAACGTGACCTCAGATACCTTAGATCCGACCAGCTGGGGTGCCGGCGGTGATGAATTGCCGGTTACCAACGGCAAGAACCCATCGCAAGAGGCGAACCTGAATCTCGCGGCAGAAGCGGTCACTCGCGGTGTTGGTGGTATGACCACGCATTGGACGTGCGCGACCCCCAAATTCAACCGCCAGGGTGAGTTGCCGAGAATCGTGAGTAACGAGCAAGAAAACTTCGATGCCTGGGAAGAGCTGTACGAGGAAGCCATGAAACTTATCGGTACTACCACCCACGCCTTCGACAAATCTATCCGCCATACCCTGGTGCTGCGTACCTTACAGAATGCGTTTAATGGCACGAACGGTAAGCCGAAACTGGACTTCGGCCCACTGCCGCTGGCGTGTCATCGTATGCCAAATCCGGATTACGTGTACTGGCACGGCACCGATGTTATTCTGGAGAAAATTTATACCAACCCGGAGCTTAAGAGCCGTTTTCGTCTGTTGACCAACCACCGTTGCACCCGCGTCGCCGTTTCCGACCATCATGCTGACGGCACTTGGACCATTGGTGCCGCGGAGGTAAAAGACTTCCTGGCCGACGTTAGCAATCATGAAGAGCAACACAGCTATGTGCAGGCAAAAATGTATGTGATCGCCGCTGGCGCTGTAGCTACCCCGCAAATCCTGTTTAACAGCGGTTTTGGCCGTAAAACCACGACGTCTATGACCACCTCTATGCTGCCGACCATTCACAATCTGGGCACCCACATTACCGAACAACCCATGTCCTTTTGTCAGATCGTATTGAAGACCTCTTTGCTTAAGACGATTCCGAACAACCCGTATAACCTGAGCTGGTGGAAAGAAAAGGTAGATTACCAGAAGGCAAAGCACCCGGAGGATCCGCTGAAGTTCCCGTTTCGTGACGGTGAACCGCAAGTTACCAAGCTGTTCACGAAGGACAAGCCGTGGCACGCACAGATTCACCGCGACGCTTTTAGCTACGGTGCGGTGGCAGAGACGATCGACACCCGTACCATCGTTGATTTCCGCTTTTTCGGTTACGTGAAACCGGAAGCGACCAATCGTATCGATTTCGAAACCAAGTATGAAGATGGCTACGGCATGCCTCAGCCGACCTTTACCTTCCGTATGTCCGACGACGATAAGACGCGTGCACACGATATGATGACGGATATGTGCAACGTGGCGAAAGAGTTGGGCGGCTGGCTGCCGGGTAGCGAACCGCAATTTATGGCGCCGGGCCTGGCATTGCATTTGGGCGGCACAGTTCGTGCAGGCCTGGACTCCAAAGAGCACGTTGCGGACACCAACTGCAAAGTCTACAATTTTTCGAACCTGTATGTTGGTGGTAATGGCGTTATTCCGACCGGTTACGCTGCGAATCCGACTCTGACCAGCATGGGTTACGCAATTCGCAGCGCACGTAGCATCATCAACGCGTTGGAGAGCGCGAAG

Table S2. Specific activity and kinetic parameters of PsPOx.

| POxs | Sequence identity (%) |
| --- | --- |
| AnPOx | 45.61 |
| TmPOx | 46.23 |
| PcPOx | 53.24 |
| LsPOx | 54.96 |

Table S3. The IC_50_ of PsPOx against three human pancreatic carcinoma cell lines (PANC-1, AsPC-1, and BxPC-3) and one normal human pancreatic epithelial cell line (hTERT-HPNE)

| Cell typs | IC_50_ (ng/mL) |
| --- | --- |
| PANC-1 | 257.8 |
| AsPC-1 | 195.2 |
| BxPC-3 | 176.2 |
| hERT-HPNE | 195.6 |

Figure S1. SDS-PAGE analysis of PsPOx expression. Lane M, protein molecular weight marker. Lane 1, soluble fraction (supernatant) of the recombinant strain after ultrasonication. Lane 2, insoluble fraction (pellet) of the recombinant strain after ultrasonication, containing inclusion bodies. Lanes 4–8, purified PsPOx eluted from the Ni-NTA column.

Figure S2. Kinetic parameters of PsPOx towards D-glucose, D-mannose, D-galactose and D-xylose.
